# Dynamic multidetector computed tomography findings of hepatocellular carcinoma of hepatitis B virus-positive and -negative patients

**DOI:** 10.1186/1470-7330-14-9

**Published:** 2014-04-22

**Authors:** Senem Senturk, Bulent Cetin, Mustafa Cengiz, Aslan Bilici, Selver Ozekinci

**Affiliations:** 1 Radiology Department, Istanbul Medeniyet University Göztepe Education and Research Hospital, Istanbul 34722, Turkey; 2 School of Medicine, Department of Internal Medicine, Division of Oncology, Gazi University, Ankara, Turkey; 3 School of Medicine, Department of Internal Medicine, Division of Gastroenterology, Gazi University, Ankara, Turkey; 4 School of Medicine, Radiology Department, Dicle University, Diyarbakir 21080, Turkey; 5 School of Medicine, Pathology Department, Dicle University, Diyarbakir 21080, Turkey

**Keywords:** Multidetector computed tomography, Hepatocellular carcinoma, Hepatitis B virus, Dynamic imaging

## Abstract

**Background:**

The objective of this study was to retrospectively investigate and compare multidetector computed tomography findings of hepatocellular carcinoma (HCC) in hepatitis B virus (HBV)-positive and -negative patients.

**Methods:**

Triphasic (arterial, portal venous, and delayed phases) dynamic multidetector computed tomography (CT) was performed in 83 patients with HCC, 48 of whom were HBV-positive. The diagnosis of HCC was established with typical CT imaging findings (68 patients) or histopathological evaluation (15 patients). Distribution of solitary, multiple, and diffuse HCC, portal/hepatic vein thrombosis, metastasis, and patients with high alpha-fetoprotein levels in the HBV-positive and -negative groups were compared using the Kolmogorov–Smirnov test. Lesion size, alpha-fetoprotein levels, arterial, portal, delayed enhancement, and washout of lesions were compared using the Student’s *t*-test.

**Results:**

Hypervascular tumors were observed in 72 (87%) patients, and hypovascular tumors were found in 11 (13%) patients. The mean alpha-fetoprotein value of HBV-positive patients with HCC was significantly higher than the mean alpha-fetoprotein value of HBV-negative patients (*P* < 0.05). Portal/hepatic vein thrombosis and metastasis were more frequently observed in HBV-positive patients (*P* < 0.05). The frequencies of solitary, multiple, and diffuse lesions in HBV-positive and -negative patients were not significantly different (*P* > 0.05). The mean diameters, arterial, portal, and delayed phase attenuations, and washout of HCC were not significantly different (*P* > 0.05).

**Conclusions:**

Multidetector CT imaging findings of HCC in HBV-positive and -negative patients are alike. Portal/hepatic vein thrombosis and metastasis are more frequently observed in HBV-positive patients. Alpha-fetoprotein levels are higher in HBV-positive patients.

## Background

Hepatocellular carcinoma (HCC), which is the most common primary liver tumor, accounts for 85–90% of primary liver cancers [1]. It is the third most common cause of cancer death and the fifth most common cancer worldwide. HCC is generally associated with chronic parenchymal liver disease, and the major risk factor for the development of HCC is cirrhosis of the liver [2]. Emerging evidence suggests that the etiology of many cases of HCC is multifactorial, including both viral infections and non-viral, environmental, and dietary exposures. Chronic hepatitis B virus (HBV) infection is the primary risk factor for the future development of HCC worldwide [3]. However, there are other important factors that may contribute to the pathogenesis of HCC. In addition to chronic infection by HBV and C (HCV) viruses, an increased body mass index and diabetes with the subsequent development of non-alcoholic steatohepatitis (NASH) represent significant risk factors for HCC. Other non-viral causes of HCC include iron overload syndromes, alcohol use, tobacco use, oral contraceptive use, and aflatoxin exposure [4].

HCC may be solitary, multifocal, or, less frequently, diffusely infiltrative. Fatty change can be seen in up to 35% of HCCs [5]. The major diagnostic techniques for HCC include serum markers, various imaging modalities, and histologic analysis. Triphasic dynamic computed tomography (CT) or magnetic resonance imaging (MRI) with arterial, portal venous, and delayed phase imaging is considered to be the primary approach for the diagnosis of HCC. Hepatocellular carcinomas are generally hypervascular and are enhanced with the highest degree of contrast during the arterial phase. Arterial phase imaging is effective in the detection of HCC, and dynamic CT, including the arterial phase, is essential for the detection of HCC [6-9]. Portal venous phase imaging is useful for detecting hypovascular liver tumors, such as metastatic tumors from the colorectum, because the liver parenchyma is enhanced maximally during this phase [10]. For the detection of HCCs, portal venous phase imaging is not sensitive because the tumors often show attenuation similar to that of the enhanced liver parenchyma, thus resulting in decreased tumor conspicuity during this phase [7,9]. However, some hypovascular HCCs may be detected only on portal venous phase images or may be depicted more conspicuously during this phase than the arterial phase. Portal venous imaging is also useful for assessing portal venous complications such as tumor thrombus [8]. Hepatocellular carcinomas are often more conspicuous on delayed phase images than on portal venous phase images, and detectability and characterization are improved by adding delayed phase imaging to the biphasic CT examination [7-9,11]. Capsule enhancement on delayed phase improves the rates of detection of HCC [12].

Although the dynamic CT findings of HCC are well defined, there are few studies to compare imaging findings of HCCs of different etiologies [13,14]. Kim et al. compared clinical and radiological findings of HCCs in patients with chronic HBV and chronic HCV infections. They found that lesions in patients with HBV were more likely to be multifocal, larger than 5 cm, in non-nodular shape, with atypical enhancement pattern, and in association with portal vein thrombosis and bile duct invasion [13]. The study by Butt et al. revealed that larger tumor size, shorter duration between diagnosis of cirrhosis and HCC, and concomitant diabetes mellitus were significant factors associated with viral marker-negative HCC [14]. However, they did not compare imaging findings of viral marker-positive and -negative HCCs. To our knowledge, the dynamic CT imaging findings of HCCs of HBV-positive and HBV-negative patients have not been compared. We undertook this study to assess and compare the triphasic, dynamic, multidetector CT (MDCT) imaging findings of the HCCs of HBV-positive and HBV-negative patients.

## Results and discussion

The risk factors of HBV-negative patients for HCC were alcohol use (5 patients), tobacco use (17 patients), NASH (3 patients), diabetes and hepatosteatosis (9 patients), and hemochromatosis (1 patient). Eleven patients had no risk factors. The risk factors of HBV-negative patients are listed in Table 1.

**Table 1:** Risk factors of hepatitis virus negative HCCs

**Risk factor**	**No of patients (%)**
Alcohol use	5
Tobacco use	17
NASH	3
Diabetes and hepatosteatosis	9
Hemochromatosis	1
No risk factor	11

NASH, non-alcoholic steatohepatitis.

Thirty-four (71%) HBV-positive patients and 24 (68%) HBV-negative patients had elevated alpha-fetoprotein values. The mean alpha-fetoprotein level of HBV-positive and -negative patients was 460 ± 80 ng/ml and 369 ± 66 ng/ml, respectively. The mean alpha-fetoprotein value of HBV-positive patients with HCC was significantly higher than the mean alpha-fetoprotein value of HBV-negative patients (*P* < 0.05). However, the frequency of elevated alpha-fetoprotein was not significantly different between two groups (*P* > 0.05). The results are summarized in Table 2.

**Table 2:** The frequency of elevated AFP values and mean AFP values of HCC in HBV positive and negative patients

**Patient group**	**No of patients**	**No of cases with elevated AFP (%)**	**Mean AFP (ng/mL)**
HBV positive	48	34 (71%)	460±80
HBV negative	35	24 (%68)	369±66
Total	83	58 (70%)	

AFP, alpha-fetoprotein; HBV, hepatitis B virus.

**Figure 1:**
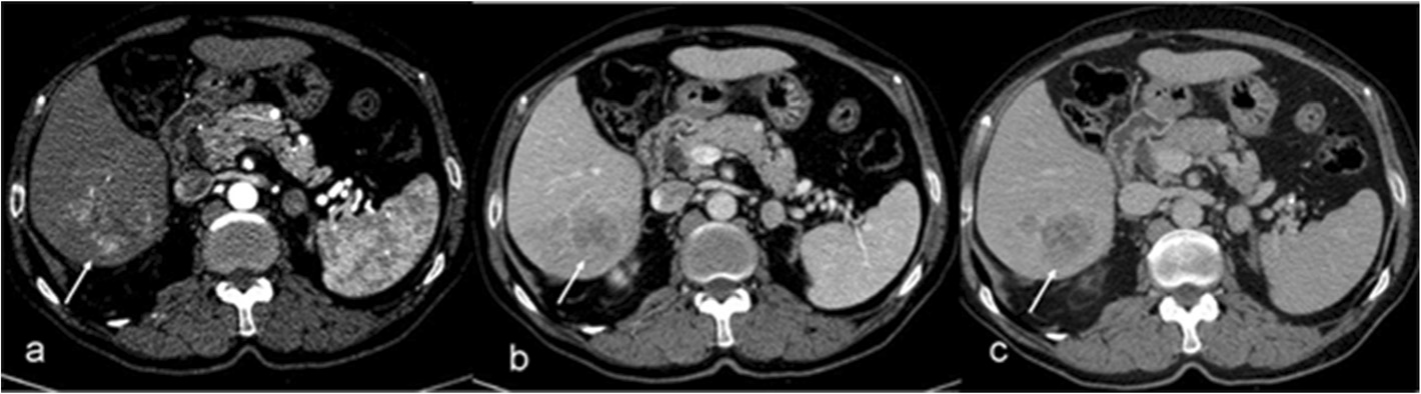
**Hypervascular hepatocellular carcinoma (arrows).** The lesion is hyperdense on the arterial phase **(a)**; however, it washes out and becomes iso-hypodense on the portal phase images **(b)** and hypodense on the delayed phase images **(c)**.

**Figure 2:**
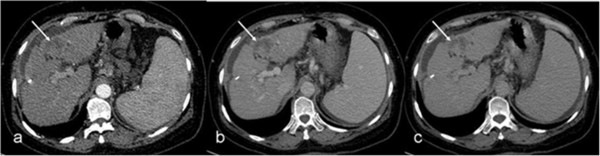
**Hypovascular hepatocellular carcinoma (arrows).** The lesion is hypodense and remains unenhanced on arterial **(a)**, portal **(b)**, and delayed **(c)** images.

**Figure 3:**
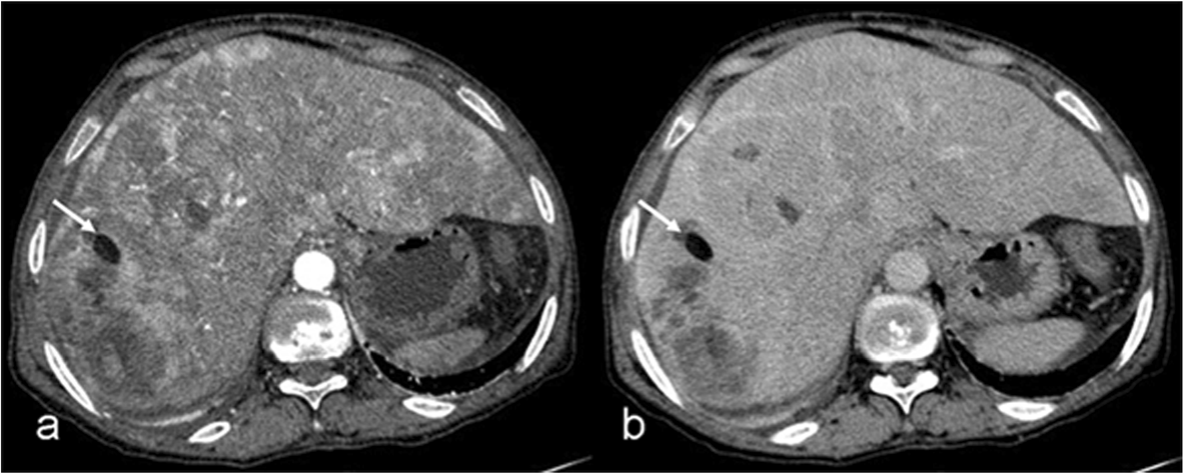
**Diffusely infiltrative hepatocellular carcinoma contains nodular fat (arrows).** The large lesion invading both lobes of the liver heterogeneously enhances on the arterial phase **(a)**, and becomes isodense with focal hypodense areas on the portal venous phase **(b)**.

All patients with HCC had at least one of the following CT findings that showed evidence of chronic parenchymal liver disease: irregular liver contours, hepatic volume loss and volume redistribution, coarse nodular liver parenchyma. Cirrhosis was confirmed histopathologically in 56 patients (67%).Seventy-two patients (87%) had hypervascular tumors, which were hyperattenuating on the arterial phase. Forty-two (87.5% of HBV-positive cases) patients with hypervascular tumors were HBV-positive and 30 (85.7% of HBV-negative cases) were HBV-negative. They were iso-hypoattenuating on the portal venous and delayed phases (Figure 1). Four lesions (6%) could only be depicted on the arterial phase. Sixty-eight (94%) hypervascular lesions were hypoattenuating (showing washout) on delayed phase images. Twenty-two (30%) hypervascular lesions showed capsule enhancement on the delayed phase. Biopsy was not required for hypervascular tumors over 2 cm that washed out or showed capsule enhancement on the delayed phase. HCC diagnosis was confirmed histopathologically by biopsy in eight lesions of 1–2 cm. Eleven (13%) patients, 7 of whom were HBV-positive, had hypovascular tumors (Figure 2). Four (36%) patients had hypovascular HCC lesions that showed capsule enhancement on delayed phase images. These patients, who had tumors > 2 cm, also had elevated alpha-fetoprotein levels, and biopsy was not required. Seven patients with hypoattenuating lesions were diagnosed by biopsy.The typical CT findings of HCC were observed in 39 (81%) of HBV-positive patients, and 29 (83%) HBV-negative patients. Fatty metamorphosis was depicted in four patients (5%), three of which were HBV-positive (Figure 3).

**Figure 4:**
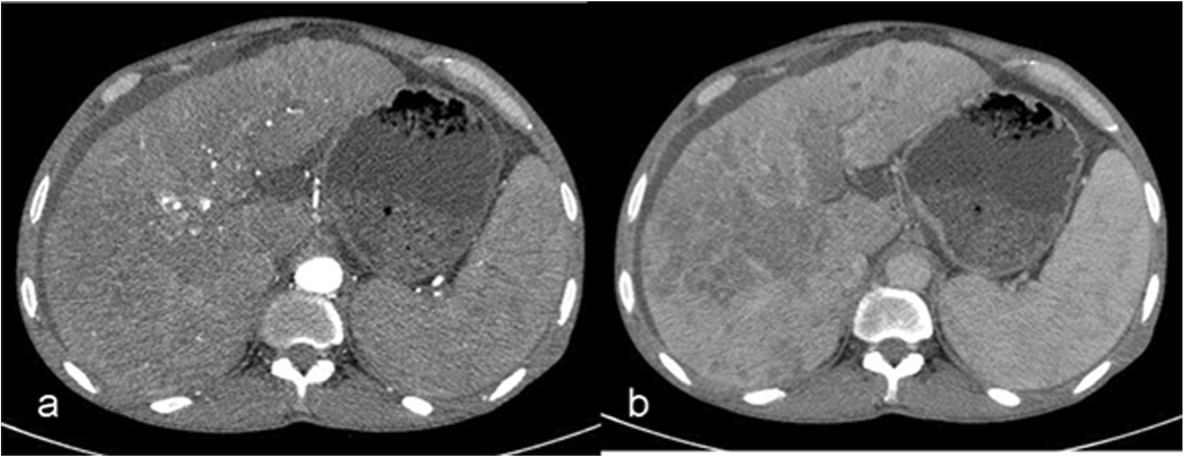
**Diffusely infiltrative hepatocellular carcinoma.** The heterogeneous lesion invades both lobes of the liver and mildly enhances on the arterial phase **(a)**. The portal venous phase image **(b)** shows a hypodense, infiltrative lesion with multiple small hypodense satellite nodules.

**Figure 5:**
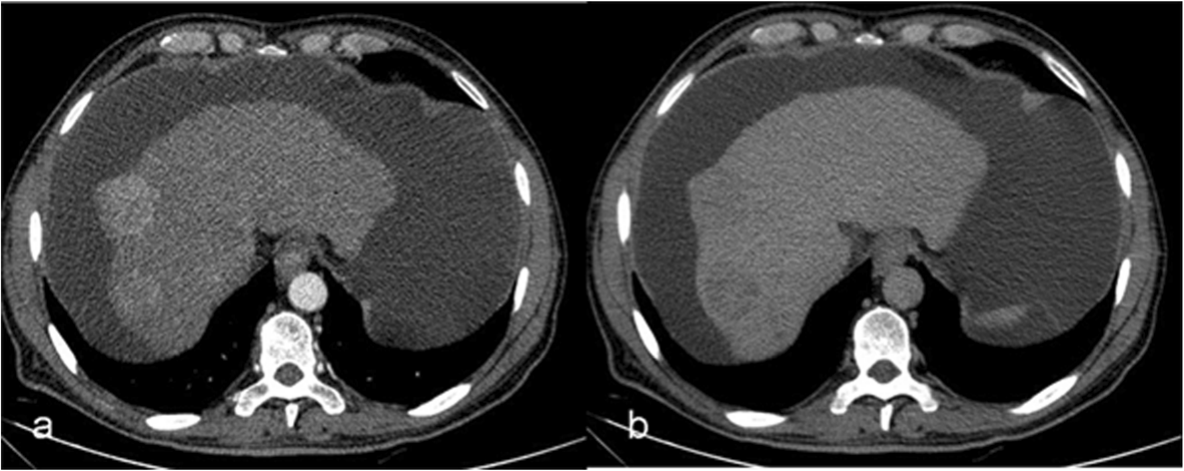
**Multiple hepatocellular carcinomas.** Two lesions in the right lobe of the liver are hyperdense on the arterial phase **(a)**; however, they wash out and become hypodense on the delayed phase image **(b)**. The patient also had two lesions in the left lobe of the liver (not shown).

The frequency of solitary lesions, multiple lesions, and diffuse lesions in HBV-positive and -negative patients are shown in Table 3. The difference was not statistically significant (*P* > 0.05). Figure 4 shows the CT images of a patient with diffusely infiltrative HCC and Figure 5 reveals multiple HCCs.

**Table 3:** The frequency of solitary lesions, multiple lesions, and diffuse lesions in HBV positive and negative patients

**Patient group**	**No of solitary lesion (%)**	**No of multiple lesion (%)**	**No of cases with diffuse HCC (%)**	**Total**
HBV positive	22 (46%)	14 (29%)	12 (26%)	48
HBV negative	12 (34%)	12 (34%)	11 (32%)	35

HBV, hepatitis B virus.

**Figure 6:**
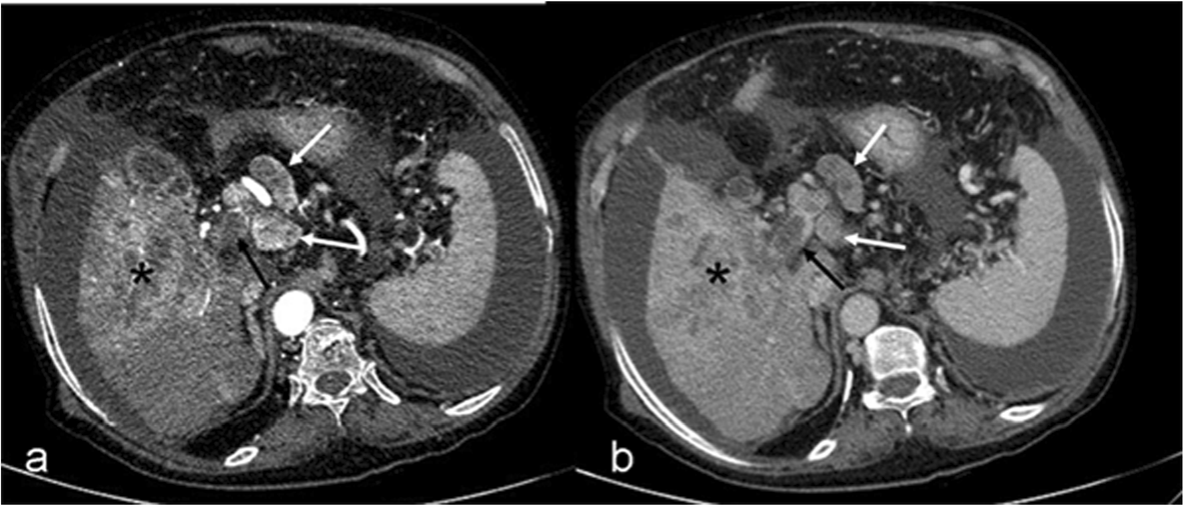
**Portal vein thrombosis (black arrows) and periportal-paraceliac lymphadenopathies (white arrows).** On the arterial phase **(a)**, lymphadenopathies are hypervascular similar to the hepatocellular carcinoma in the right lobe of the liver (asterisk). The hepatocellular carcinoma and lymphadenopathies wash out on the portal venous phase **(b)**.

The frequencies of portal/hepatic vein thrombosis and metastasis in HBV-positive and -negative patients are shown in Table 4. Portal/hepatic vein thrombosis and metastasis were more frequently observed in HBV-positive patients (*P* < 0.05). Figure 6 shows a HCC lesion with portal thrombosis and periportal metastatic lymphadenopathy.

**Table 4:** The frequency of portal and/or hepatic venous thrombus and metastasis in HBV positive and negative patients

**Patient group**	**No of patients with portal and/or hepatic venous thrombus (%)**	**No of patients with metastasis (%)**
HBV positive	20 (%42)	15 (%31)
HBV negative	8 (%23)	4 (%11)

HBV, hepatitis B virus.

All patients with portal/hepatic vein thrombosis had a tumor thrombus that revealed CT enhancement patterns similar to HCCs. Non-tumoral, acute thrombus was not observed; however, three patients, two of whom were HBV-positive, had cavernous transformation of the portal vein revealing a history of chronic bland thrombus.

The most common metastatic site was lymph nodes in both HBV-positive and -negative patients. Periportal and para-aortic nodal metastases were observed in 12 patients, 10 of whom were HBV-positive. Four HBV-negative patients had metastases; two patients had lymph node metastases, one had adrenal metastasis, and one had bone metastases. Fourteen HBV-positive patients had extralymphatic metastases: 5 adrenal, 2 common bile duct, 2 lung, 3 bone, 1 renal, and 1 peritoneal.

The maximum diameter of the HCC lesions ranged from 10 to 189 mm. The mean diameter of the HCC lesions of HBV-positive patients was 77 ± 56 mm, and the mean diameters of the HCC lesions of HBV-negative patients was 65 ± 49 mm. The difference was not statistically significant (*P* > 0.05).

The mean arterial, portal, and delayed phase attenuations, and the washout of HCC lesions of HBV-positive and -negative patients, are shown in Table 5. The differences between attenuations and washout of HCCs in HBV-positive and HBV-negative patients were not statistically significant (*P* > 0.05).

**Table 5:** Mean arterial, portal, delayed phase attenuations and washout of HCC lesions of HBV positive and negative patients

**Patient group**	**Arterial phase attenuation (mean HU)**	**Portal phase attenuation (mean HU)**	**Delayed phase attenuation (mean HU)**	**Washout (mean HU)**
HBV positive	100±20	74±12	60±12	39±17
HBV negative	99±22	73±10	62±11	36±20

HBV, hepatitis B virus; HU, Hounsfield unit.

Continuous inflammation and hepatocyte regeneration in cirrhosis may lead to chromosomal damage that initiates hepatic carcinogenesis. In HBV-associated carcinogenesis, viral factors are likely to be involved, and HCC may occur in patients without significant background liver fibrosis. There is strong epidemiological association between chronic HBV infection and HCC. Previous studies showed that larger tumor size, shorter duration between the diagnosis of cirrhosis and HCC were significant factors associated with viral marker-negative HCC [14].

In this study, there was no difference between imaging appearance (mean arterial, portal, and delayed phase attenuations and washout degrees) of HCC lesions in HBV-positive and -negative patients. Contrast enhancement patterns, and the size and number of lesions were alike. Thus, in this study, we did not observe a difference between the MDCT imaging findings of the HCCs of HBV-positive and -negative patients. To our knowledge, the MDCT imaging findings of HBV-positive and -negative patients has not been compared in previous studies.

We showed that tumor thrombus and metastases were more common in HBV-positive patients. Vascular invasion and tumor thrombus may lead to extrahepatic spread of tumor cells; extensive resection and adjuvant treatment may then be indicated. Since the occurrence of vascular thrombus changes the surgical plan, HBV-positive patients should be carefully evaluated to rule out tumor thrombosis. In case of tumor thrombosis and cavernous transformation of the main portal vein, liver transplantation may not be possible.

Alpha-fetoprotein has been used as a serum marker for HCC for many years. Some patients with cirrhosis and/or hepatic inflammation can have an elevated alpha-fetoprotein, even without the presence of a tumor. In previously published studies, the test has been shown to have a sensitivity of 39–65%, a specificity of 76–94%, and a positive predictive value of 9–50% for the presence of HCC [15]. Trevisani et al. defined an alpha-fetoprotein level of 16 ng/ml as the threshold that maximized sensitivity and specificity for the diagnosis of HCC [16]. Seventy per cent of our patients had an alpha-fetoprotein value > 16 ng/ml. The frequency of elevated alpha-fetoprotein was not significantly different between HBV-positive and -negative patients. However, the mean alpha-fetoprotein value of HBV-positive patients was significantly higher than the mean alpha-fetoprotein value of HBV-negative patients. Metastases were more frequent in HBV-positive patients. Moreover, HBV-positive patients had HCCs with a larger mean diameter, which might have caused higher alpha-fetoprotein values. However, it should be noted that the size of lesions ranged from 10 mm to 189 mm, and that the difference between the mean lesion size of the two groups was not significant due to such a wide range.

Ishikawa suggested that HBV-related HCC may occur in younger patients with mild inflammation and fibrosis than in patients with HCV-related HCC [3]. However, in many cases of HBV-related HCC, the tumor stage is advanced, presenting an undifferentiated cancer type with portal vein infiltration, suggesting that HBV-related HCC has a worse prognosis and a more refractory character than HCV-related HCC [3]. Moreover, HBV-related HCC is infiltrative, showing multinodular growth, unlike HCV-related HCC [17]. HBV-related HCC is often accompanied by portal vein tumor thrombus [3]. We also observed portal/hepatic vein thrombosis and metastasis more frequently in HBV-positive patients, supporting that advanced disease stage is more common in HBV-related HCC. In this study, we could not compare HBV-positive and HCV-positive patients, because most HCV-positive patients in this study were also HBV-positive. These patients were excluded from the study group. We found only five HCV-positive patients without HBV. Because of this small number, these patients were also excluded from the study group.

Our study has some limitations. We had a small sample size and limited histopathologic correlation. The major risk factors for HBV-negative HCCs could not be clearly identified. Some patients had no risk factors, whereas some had more than one risk factor. HBV-negative patients were composed of a heterogeneous patient group. Another limitation was the lack of histopathological diagnosis of cirrhosis in some patients; however, all patients showed CT evidence of chronic parenchymal liver disease. Prospective studies with larger patient cohorts are necessary to clearly define the risk factors of HBV-negative patients, and compare the CT imaging findings and clinical outcomes of these patients. Because of the retrospective design of the study, we could not compare the clinical outcomes of HBV-positive and -negative patients.

## Conclusion

We have shown that the MDCT imaging findings of HCCs of HBV-positive and -negative patients were alike. In this study, we observed that the frequency of elevated alpha-fetoprotein was not different between HBV-positive and -negative patients; however, the mean alpha-fetoprotein value of the HBV-positive patients was higher. Portal/hepatic vein thrombosis and metastasis were more frequent in HBV-positive patients.

## Methods

### Patients and hepatocellular carcinoma diagnosis

A retrospective review of the computer-based radiology archive of Dicle University was performed to determine patients with HCCs who had undergone dynamic liver MDCT. HCCs with either typical dynamic CT imaging findings or histopathologic diagnosis were included in the study. We excluded patients with either a positive serology for HCV or a technically inadequate CT examination. We excluded three patients because of technically inadequate examinations with artifacts. We found that 65 consecutive HBV-positive patients with HCCs had undergone technically adequate dynamic MDCT between January 2007 and June 2009. Seventeen patients were excluded from the study group because they were also HCV-positive. Since HCCs were much more common in patients with HBV, we had to review the radiology archive for a longer period to find a large-enough number of HBV-negative patients. Forty consecutive HBV-negative patients with HCCs had undergone technically adequate dynamic MDCT between January 2007 and December. Due to HCV infection, five patients were excluded.

The study group consisted of 83 patients (57 men, 26 women; age range, 49–85 years; mean age, 66 years) with HCCs either diagnosed by MDCT findings or histopathologically by biopsy. Forty-eight patients were HBV-positive and 35 patients were HBV-negative. The risk factors of HBV-negative patients were searched from their hospital records. The alpha-fetoprotein values of all patients were recorded. An alpha-fetoprotein level of 16 ng/ml was used as the threshold.

HCC lesions greater than 2 cm in diameter can be diagnosed non-invasively, based on radiographic criteria in patients with cirrhosis [18]. Histologic confirmation by biopsy is not mandatory owing to the excellent diagnostic accuracy of imaging criteria and the 10–20% false-negative rate from histologic samples [19]. Recent studies concluded that in cirrhotic patients and patients with chronic hepatitis B, nodules greater than 1 cm in diameter that reveal the typical features of HCC on dynamic profile (arterial hypervascularity with washout in the portal or delayed venous phase) can be diagnosed as HCC by using a single imaging modality [20]. Histologic diagnosis is recommended if the vascular pattern is not characteristic for HCC on imaging modalities, to establish the diagnosis [20].

In this study, the diagnosis of HCC was based on imaging findings in 68 patients, 39 of whom were HBV-positive. Image analysis was performed by a radiologist (SS) who had 6 years’ experience in abdominal imaging. Sixty-eight patients had HCCs over 2 cm in diameter that revealed typical dynamic CT findings (either arterial hypervascularity with washout in the portal or delayed venous phase or typical capsule enhancement on delayed phase). Since CT findings were inconclusive in 15 patients (8 patients with hypervascular lesions of 1–2 cm, 7 patients with hypovascular lesions > 2 cm), ultrasound-guided fine needle aspiration biopsy (FNAB) and histopathologic diagnosis was required. Two HBV-positive patients (13%) required repeated FNAB, because the initial procedure was not diagnostic. Histopathological analysis was performed by a pathologist (SO) who had 11 years’ experience in liver pathologies. Radiologic-pathologic correlation was performed by the same radiologist and pathologist who established diagnosis of HCC.

To evaluate abdominal metastasis, portal phase CT images covering the whole abdomen from the diaphragm to the symphysis pubis were investigated. When HCC diagnosis was established, chest CT and bone scintigraphy were also performed to evaluate further metastases.

### Computed tomography scanning protocol and image assessment

All studies were performed on a CT scanner with 64 detectors (Brilliance CT 64-channel scanner; Philips Medical Systems Inc., Cleveland, OH, USA). An 18–20 gauge angiocatheter was introduced into the antecubital vein of each patient. The administration rate was 4 ml/s; 300 mg/ml of 100–150 cc (1–2 ml/kg) nonionic iodine solution was used as the contrast medium. Dose parameters were 250–300 mAs and 120 kV. The scan parameters were as follows: reconstruction interval 1.5, slice thickness 3 mm, pitch factor 0.79–1.11, rotation time 0.5 s, collimation 64 × 0.625.

**Figure 7:**
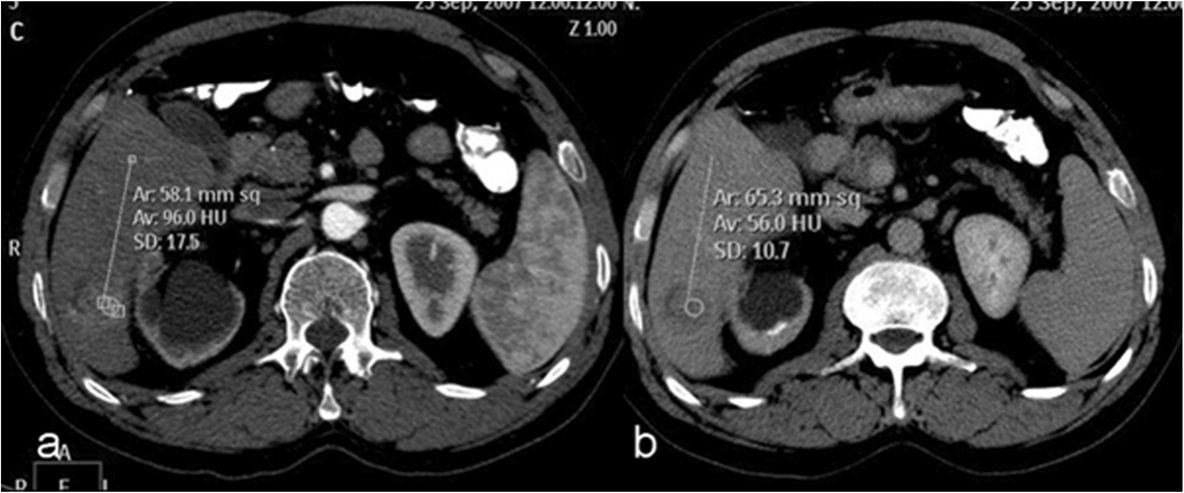
Measurements of computed tomography (CT) attenuations (in Hounsfield units) of a hypervascular hepatocellular carcinoma on arterial (a) and delayed (b) phase CT images.

Three-phase, contrast-enhanced CT of the liver was performed during the hepatic arterial phase, portal venous phase, and delayed phase. Arterial phase imaging was performed 20 s after the start of the contrast material injection. The delay time was 50 s for portal venous phase imaging and 3 min for delayed phase imaging. All studies were started at the top of the liver and proceeded in a cephalocaudal direction; arterial phase and delayed phase, contrast-enhanced helical scans of the entire liver were obtained. The whole abdomen, from diaphragm to symphysis pubis, was scanned at the portal venous phase. Patients were asked to hold their breath during scanning.Arterial enhancement, portal venous enhancement, and washout of lesions were measured for each lesion. Three measurements were performed on transverse, 3-mm reconstructed images, with no intersectional gap for each phase set. Mean CT values in Hounsfield units (HUs) in the HCC lesions were measured in all patients on the workstation (Extended Brilliance Workspace; Philips Healthcare, Best, The Netherlands) by using a circular region-of-interest cursor ranging in size from 5 to 30 mm in diameter. Measurements were performed on the arterial, portal, and delayed phase images (Figure 7). For heterogeneously enhancing lesions, arterial phase measurements were performed from the most enhancing portion, and on portal venous and delayed phases the same portion of the lesion was used for the measurements. When there was more than one lesion, mean values were calculated for each patient.

The number of solitary, multiple, and diffuse HCC lesions was determined. Whole-abdomen images of the portal phase were reviewed to search for portal/hepatic vein thrombosis and metastasis. The maximum diameters of all lesions were measured. When there was more than one lesion, mean values were calculated for each patient.

### Statistical analysis

The SPSS software package, version 11.01 (SPSS Inc., Chicago, IL, USA), was used for data analysis and a *P* value of 0.05 was considered statistically significant. Data were presented as mean ± standard deviation.

The frequency of elevated alpha-fetoprotein was calculated as a percentage for HBV-positive and -negative patients. The alpha-fetoprotein values of the two groups were compared using the Student’s *t*-test. The distribution of elevated alpha-fetoprotein levels in HBV-positive and -negative patients were compared using the Kolmogorov–Smirnov test.

The frequencies of solitary, multiple, and diffuse HCCs, and the portal and hepatic venous thrombosis and metastasis were determined in HBV-positive and -negative patients. The distribution of lesion numbers, thrombosis and metastasis in the two groups (HBV-positive and -negative patients) were compared using the Kolmogorov–Smirnov test.

Arterial enhancement, portal venous enhancement and washout values of lesions were compared using the Student’s *t*-test. The washout was calculated by subtraction of delayed phase HU values from arterial phase HU values (washout (HU) = arterial phase enhancement (HU) - delayed phase enhancement (HU)).

## Competing interests

The authors declare that they have no competing interest.

## Authors’ contributions

SS carried out CT evaluation and measurements, SS and AB participated in the statistical analysis and drafted the manuscript. SS, BC and MC performed literature search and created the study design. SO performed histopathological analysis and drafted the manuscript. All authors read and approved the final manuscript.
